# Comparative Assessment of Cartilage Quality in Human Induced Chondrocytes (hiCHOs) and Primary Articular Chondrocytes (hACs) Following Fibronectin-Based Selection

**DOI:** 10.1177/19476035261458719

**Published:** 2026-06-08

**Authors:** Giorgia Mazzini, Margo Tuerlings, Vicki van der Stap, Ilja Boone, Rachid Mahdad, Yolande F.M. Ramos, Ingrid Meulenbelt

**Affiliations:** 1 Department of Biomedical Data Sciences, Section Molecular Epidemiology, 4501 Leiden University Medical Center, Leiden, The Netherlands; 2 Department of Orthopaedics, 4499 Alrijne Hospital, Leiderdorp, The Netherlands

**Keywords:** chondrocytes, articular chondroprogenitor cells, fibronectin, human induced pluripotent stem cells, chondrogenesis

## Abstract

**Background:**

Cell-based cartilage repair is limited by loss of chondrogenic potential during *in vitro* expansion. Fibronectin (FN)-based selection may enrich progenitor-like cells with better matrix-forming ability, but its long-term effect on ECM production by hiPSC-derived chondroprogenitors (hiCPCs), their derived chondrocytes (hiCHOs), and primary articular chondrocytes (hACs) from lesioned or preserved areas remains unclear.

**Methods:**

We here compared cartilage ECM formation by hiCPCs/hiCHO’s and hACs, with or without FN selection, over extended passaging using 3D organoids. Histology and quantitative image analysis were performed to assess tissue quality across passages.

**Results:**

Chondrogenicity in hiCPCs transiently improved early-stage ECM production upon FN-based selection, but accelerated loss of chondrogenicity was seen in later passages. This was evidenced by diminished matrix staining and structural degradation from passage 4 onward. In contrast, hACs from both preserved and lesioned cartilage maintained stable matrix-forming ability across passages, independent of FN-based selection.

**Conclusion:**

FN-based selection does not preserve long-term chondrogenicity in hiCHOs and may even impair it during extended culture. In contrast, primary cell sources, regardless of cartilage integrity, demonstrate greater and robust chondrogenic capacity without requiring progenitor enrichment. These findings highlight the intrinsic ability of hACs and question the utility of FN-based selection in cartilage tissue engineering.

## Introduction

Osteoarthritis (OA) is a degenerative joint disease characterized by progressive breakdown of articular cartilage, leading to joint pain, stiffness, and loss of mobility. Despite its high prevalence and social and economic burden to society, there is no therapy available yet to prevent or slow down OA pathophysiology, except for total joint replacement surgery at end-stage disease. One of the primary risk factors for OA is joint tissue overloading or trauma,^1,2^ largely because articular cartilage has a limited ability to repair itself.^3^ Tissue engineering and cell-based therapies with primary autologous cells are being actively developed as strategies to restore cartilage integrity.

Human primary articular chondrocytes (hACs) are commonly used for cartilage repair in a procedure called autologous chondrocyte implantation (ACI). This is because hACs are known to produce high quality neo-cartilage *in vitro* that is very similar to that of autologous cartilage as reflected by Alcian Blue staining of the deposited glycosaminoglycans (sGAGs) and the cellular epigenetic profile.^4^ Nevertheless, a well-recognized limitation of hACs is their gradual dedifferentiation and loss of chondrogenic potential during *in vitro* passaging, with passage 2 being the standard assuring high quality cartilage.^5^ As a result, ACI is applied primarily in patients with small superficial cartilage defects. Over the past decades, a population of endogenous articular cartilage progenitor cells (ACPCs) has been identified in both animals and humans.^6-8^ Williams et al.^6^ demonstrated that these ACPCs can be isolated from articular cartilage-derived chondrocytes using a fibronectin (FN) adhesion assay. These ACPCs exhibit accelerated proliferative capacity *in vitro* as compared to hACs while maintaining the ability to produce high-quality cartilage matrix based on Toluidine Blue and Safranin O staining.^7,9^ Despite these advancements, the application of hACPCs as an alternative of hACs in regenerative treatments, remains debated. For example, Jovic et al.^10^ reported no clear regenerative benefit from FN adhesion assay-derived progenitors obtained from nasoseptal cartilage, while Vinod et al^11^ observed that naturally migratory progenitors may retain greater chondrogenic capacity than adhesion-selected counterparts.

On a different note, human induced pluripotent stem cells (hiPSCs) have been introduced as novel scalable and non-invasive source for generating neo-cartilage. Owing to their unlimited self-renewal capacity and pluripotency, hiPSCs can be expanded indefinitely and differentiated into virtually any cell type, thereby paving the way for regenerative applications in cartilage repair. Using a step-wise protocol,^12^ hiPSCs can be differentiated into chondroprogenitor cells (hiCPCs) and subsequently into chondrocytes (hiCHOs). In our recent study,^13^ we compared the neo-cartilage produced by these hiCHOs to that derived from hACs and we found high similarity between these cell types. Even though these findings support hiCHOs as a promising alternative to hACs for cartilage regeneration strategies, the differentiation protocol from hiPSCs toward hiCHOs remains time-consuming and labor-intensive. Even more, the intermediate hiCPCs could potentially offer the advantage of being an off-the-shelf cell source for timely cartilage production. But nonetheless, their clinical utility is limited by poor expandability and a rapid decline in chondrogenic potential of hiCPCs within a few passages.^12^

To this end, the present study aims to: (1) assess the chondrogenic capacity of hACs, representing mature cartilage cells, after extensive passaging and determine whether this can be improved through hACPC selection, and (2) investigate whether FN-based selection can yield a more stable, progenitor-enriched subpopulation within the heterogeneous hiCPC pool that can maintain chondrogenic performance over extended passaging. To address these aims, we generated hAC- and hiCHO-derived 3D cartilage organoids across serial passages. We assessed cartilage quality using Alcian Blue staining, as deposition of GAG-rich extracellular matrix (ECM) is a hallmark of functional cartilage. Moreover, we determined the proliferation capacity of these cells by calculating population doubling times (PDTs), as an indicator of changes in proliferative behavior.

## Materials and Methods

### Sample Description and Ethics Approval

Primary chondrocytes were isolated from OA joints collected from total joint replacement surgery, as part of the RAAK-study.^14^ Patients' characteristics are shown in Supplementary Table S1. Ethical approval for the RAAK study was obtained from the medical ethics committee of the LUMC (P19.013), and informed consent was obtained from all patients.

An independent control hiPSC line was used in the current study. Cells were generated by a non-integrative RNA reprogramming method from skin fibroblasts of a Caucasian female (LUMC0099iCTRL04 line) by the LUMC iPSC core facility and registered at the Human Pluripotent Stem Cell Registry. Cells were characterized according to pluripotent potential and spontaneous differentiation capacity by the iPSC core facility and were karyotyped after 15 passages in culture.^15^ Approval for the generation of hiPSCs from skin fibroblasts of healthy donors is available under number P13.080.

### Cell Culture of hACs and hACs-Derived 3D Organoids

**Figure 1.**
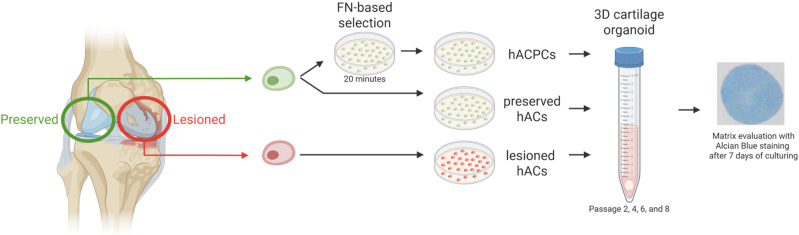
hACs experimental set-up. Primary cells were isolated from macroscopically preserved and lesioned articular cartilage of 3 human OA knee joints. Subsequently, half of the preserved cells were exposed to a fibronectin adhesion assay to capture the chondrogenic progenitor cells (hACPCs). The cells were expanded in 2D and in passage 2, 4, 6, and 8 3D cartilage organoids were created and the matrix quality was evaluated using Alcian Blue staining

Cartilage was collected from macroscopically preserved and lesioned areas of OA knee joints. The hACs were isolated from the cartilage by overnight incubation in expansion medium (DMEM (high glucose; Gibco), supplemented with 10 % fetal bovine serum (FBS; Gibco), 100 U/ml penicillin and 100 µg/ml streptomycin (Gibco), 0.5 ng/ml FGF-2 (Peprotech), with theaddition of 2 mg/ml collagenase type I. To remove undigested cartilage fragments, the medium was passed through a 70 µm strainer, leaving behind a suspension of cells. As shown in Figure 1, the preserved cell suspension was either plated to a normal culture dish or to a FN-coated dish (10 µg/mL in PBS + Ca^2+^ and Mg^2+^ for 1 h at 37 °C). The cell suspension added to the FN-coated dish was incubated for 20 minutes to allow articular chondroprogenitor cells (hACPCs) to attach. After 20 minutes, the remaining suspension was removed and the dish was refreshed with expansion medium. Cells were passaged upon reaching approximately 80% confluency and population doubling times were calculated in each passage to assess cell proliferation dynamics.

Moreover, 3D organoid cultures were created in different passages (P2, P4, P6 and P8). To create the cartilage organoids, 2.5 x 10^5^ cells in their expansion medium were added to a 15 mL Falcon tube and subsequently exposed to centrifugal forces (1200 rpm, 4 minutes).

After 24 hours, the expansion medium was replaced by chondrogenic differentiation medium (DMEM (high glucose; Gibco), supplemented with Ascorbic acid (50 μg/ml; Sigma-Aldrich), L-Proline (40 μg/ml; Sigma-Aldrich), Sodium Pyruvate (100 μg/ml; Sigma-Aldrich), Dexamethasone (0.1 μM; Sigma-Aldrich), ITS+, 100 U/ml penicillin and 100 μg/ml streptomycin (Gibco) and TGF-β1 (10 ng/ml; Peprotech), as described previously.^16^ Medium was refreshed on day 3 or 4 and on day 6 or 7. The 3D cartilage organoids were harvested on day 7 and processed for histology (Figure 1).

### Cell Culture of hiPSCs

hiPSCs were maintained in mTeSR-plus medium (STEMCELL Technologies) on Vitronectin XF-coated plates (STEMCELL Technologies). The medium was refreshed every two days, and cells were passaged in aggregates using Gentle Cell Dissociation Reagent (STEMCELL Technologies) upon reaching approximately 80% confluency.

### Stepwise Chondrogenic Differentiation of hiPSCs

Generation of induced chondroprogenitor cells (hiCPCs) was based on a protocol previously described.^12^ When hiPSCs reached 60% confluence, the culture medium was switched to mesodermal differentiation (MD) medium, composed of IMDM GlutaMAX (IMDM; Thermo Fisher Scientific) and Ham’s F12 Nutrient Mix (F12; Sigma-Aldrich) with 1 % chemically defined lipid concentrate (Gibco), 1 % insulin/human transferrin/selenium (ITS+; Corning), 0.5 % penicillin-streptomycin (P/S; Gibco), and 450 μM 1-thioglycerol (Sigma-Aldrich). Before induction of anterior primitive streak (day 0), hiPSCs were washed with wash medium (IMDM/F12 and 0.5 % P/S) and then fed with MD medium supplemented with activin A (30 ng/ml; Stemgent), 4 μM CHIR99021 (CHIR; Stemgent), and human fibroblast growth factor (20 ng/ml; FGF-2; R&D Systems) for 24 hours. Subsequently, the cells were washed again with wash medium, and paraxial mesoderm was induced on day 1, by MD medium supplemented with 2 μM SB-505124 (Tocris), 3 μM CHIR, FGF-2 (20 ng/ml), and 4 μM dorsomorphin (Tocris) for 24 hours. Before induction of early somite (day 2), cells were washed with wash medium, and then cells were fed with MD medium supplemented with 2 μM SB-505124, 4 μM dorsomorphin, 1 μM C59 (Cellagen Technology), and 500 nM PD173074 (Tocris) for 24 hours. Subsequently, cells were washed with wash medium, and for induction of sclerotome, cells (days 3 to 5) were fed daily with MD medium supplemented with 2 μM Purmorphamine (Stemgent) and 1 μM C59. To induce chondroprogenitor cells (days 6 to 14), cells were washed briefly with wash medium and fed daily with MD medium supplemented with human bone morphogenetic protein 4 (BMP-4; 20 ng/ml; Miltenyi Biotec). Monolayer-cultured hiCPC aggregates present at day 14 of the differentiation were first harvested and then washed twice with PBS, dissociated with TrypLE (Gibco-Thermo Fisher) at 37 °C, and centrifuged for 5 min at 1640 rpm. The resulting pellets were further dissociated by pipetting for being able to get as many single cells as possible before proceeding with counting and seeding the cells for either expansion or FN-based selection.

For expansion, hiCPCs were cultured as previously described.^12,17,18^ Briefly, cells were plated on culture dishes and maintained in DMEM/F-12 (GlutaMAX) supplemented with 10% fetal bovine serum (FBS), 1% ITS+, 55 μM 2-mercaptoethanol, 1% non-essential amino acids (NEAA), 1% penicillin/streptomycin, 40 μg/mL bFGF, and 50 μg/mL L-ascorbic acid 2-phosphate. Medium was refreshed every two days, and cells were passaged at approximately 70–80% confluency.

At every passage of each condition, 2.5 x 10^5^ hiCPCs were harvested and centrifuged for 5 min at 1640 rpm in order to form aggregates that were subsequently cultured and maintained in 3D suspension culture in 15 mL tubes in chondrogenic differentiation (CD) medium containing Dulbecco’s modified Eagle’s medium/F12 (Gibco), supplemented with 1% ITS+, 55 μM 2-mercaptoethanol (Gibco), 1 % non-essential amino acids (Gibco), 0.5 % P/S, L-ascorbate-2-phosphate (50 μg/ml; Sigma-Aldrich), L-proline (40 μg/ml; Sigma-Aldrich), ML329 (1 µM; CSNpharm), C59 (1 µM; Tocris), and transforming growth factor–β1 (10 ng/ml; Peprotech) for up to 28 days to obtain human induced chondrocytes (hiCHOs) while refreshing medium every 3 to 4 days. Population doubling time (PDT), defined as the average time required for a cell population to double in number, was calculated at every passage to monitor cell proliferation dynamics and assess potential differences in growth rates between conditions. PDT is commonly used as an indicator of proliferative capacity and maintenance of a stable cellular phenotype during *in vitro* expansion. 

### Histological and Immunohistochemical Staining

The 3D cartilage organoids derived from hACs or hiCHOs were fixed in 4% formaldehyde at day 7 or day 28, respectively. Subsequently, the organoids were embedded in paraffin and 5 µm sections were made. The sections were deparaffinized, rehydrated and stained for glycosaminoglycan (GAG) deposition using Alcian Blue staining (Sigma-Aldrich), with Nuclear Fast Red (Sigma-Aldrich) as a counterstain. Moreover, the sections were stained for proteoglycan-rich extracellular matrix using Safranin-O (0.2 g Safranin T (Fluka), 1 ml 100% acetic acid and 100 ml distilled water), with Fast green (0.04 g Fast Green (Chroma), 0.2 ml 100% acetic acid, and 100 ml distilled water) as a counterstain. Finally, we performed immunohistochemical staining for COL2 (AB34712) and COL1 (AB34710). Antigen retrieval was done by using proteinase K (5 µg/ml, Qiagen), followed by hyaluronidase treatment (5 mg/ml, Sigma). The sections were incubated overnight with the primary antibody, after which they were incubated with HRP secondary antibody (ImmunoLogic). Visualization was done using diaminobenzidine and a counterstain with haematoxylin was performed.

To further assess relative collagen composition, a normalized collagen ratio was calculated based on immunohistochemical staining intensities. Specifically, the proportion of collagen type I was expressed as COL1/(COL1 + COL2), using mean intensity values obtained from ImageJ analysis. This ratio provides an estimate of the relative contribution of fibrocartilage-associated (COL1) versus hyaline cartilage-associated (COL2) matrix components.

Quantification of staining intensity was performed using Fiji/ImageJ (version 1.54p). Negative high-resolution images of stained neo-cartilage sections were converted to 8-bit grayscale, and identical thresholds were applied across all images to ensure consistency. Regions of interest (ROIs) encompassing the entire tissue area were manually outlined to exclude background. Mean gray values, representing staining intensity, were measured for each ROI. Because a negative image was used, the scale was inverted: 0 corresponded to white and 255 to black, meaning that higher values reflected stronger staining intensity. For each condition and passage, at least three technical replicates were analyzed.

### Statistical Analysis

Statistical analyses were performed using SPSS version 29.0.0.0 (IBM, Armonk, NY, USA). The reported P values comparing FN-based selection condition and control were determined by applying generalized estimating equations (GEEs) and *P*-values ≤0.05 were considered significant.

## Results

### Human Primary Articular Chondrocytes (hACs)-Derived Neo-Cartilage Quality Upon Fibronectin-Based Selection

**Figure 2.**
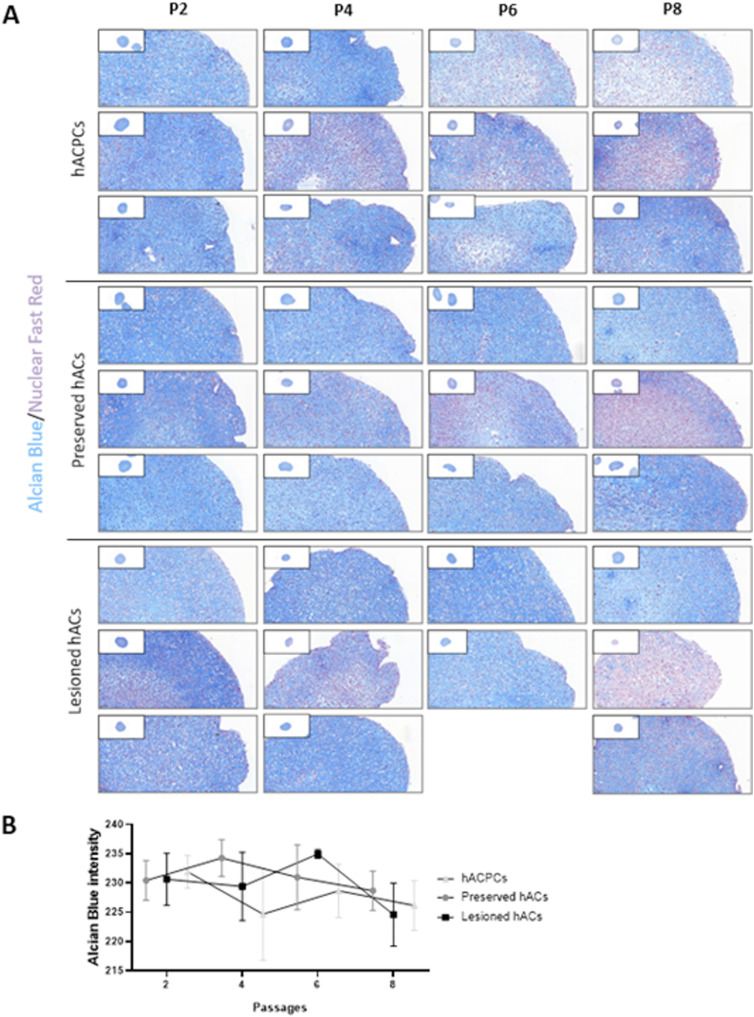
Alcian blue staining of hACPC-, preserved hAC- and lesioned hAC-derived 3D cartilage organoids in multiple passages. (A) Representative Alcian Blue/Nuclear Fast Red-stained sections. The overview images were captured at 4x magnification, while the zoomed-in images were taken at 20x magnification. (B) Quantification of Alcian Blue intensity. GEE was used to determine statistical differences (Supplementary Table S2)

HACs were isolated from both macroscopically preserved and lesioned regions of three independent human OA knee joints (Supplementary Table S1A). To enrich for hACPCs, half of the preserved hACs were selected with a FN adhesion assay, while the other hACs were treated according to our custom protocol (Figure 1).^4^ All cells (hACPCs, preserved hACs and lesioned hACs) were expanded in 2D culture and passaged upon reaching 80% confluency. To evaluate the chondrogenic potential of these cells, we created 3D cartilage organoids at consecutive passages (P2, P4, P6 and P8) and evaluated cartilage matrix deposition by Alcian Blue staining. As shown in Figure 2A, the cartilage matrix produced by hACPCs, preserved hACs, and lesioned hACs at passage 2 was highly similar showing consistent Alcian Blue (AB) intensity. Notable is also from Figure 2A and B that although variation in the staining intensity with further passaging emerged in all three cell sources, the overall reduction in Alcian Blue intensity was modest, and not significant up until passage eight (Figure 2B, Supplementary Table 2A). Together, our data show that the chondrogenic phenotype in our experimental groups was largely maintained upon passaging and that the FN adhesion assay-derived hACPCs did not outperform preserved hACs nor lesioned hACs (Figure 2B, Supplementary Table 2B).

To further assess matrix composition, COL1 staining was performed in neo-cartilage produced by hACPCs, preserved hACs, and lesioned hACs (Supplementary Figure S1, Supplementary Table S3). In line with the reported Alcian Blue results, COL1 levels remained relatively stable across passages in all conditions. When comparing cell sources, cartilage derived from lesioned hACs showed an overall higher tendency toward higher COL1 levels, which became more apparent at later passages compared to cartilage from preserved hACs and hACPCs. In contrast, cartilage from preserved hACs and hACPCs was comparable, with no significant differences observed, indicating that FN-based selection did not clearly affect COL1 deposition in these cells.

**Figure 3.**
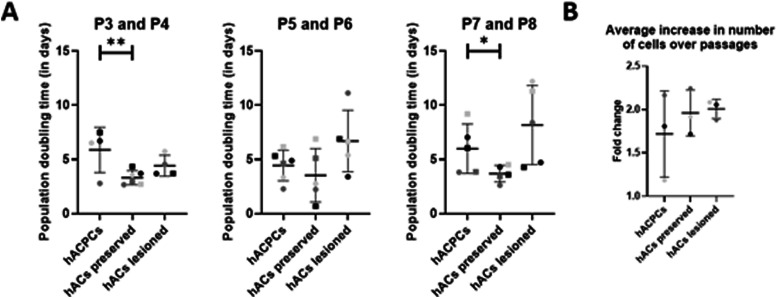
Population doubling time and increased cell numbers per passage of hACPCs, preserved hACs and lesioned hACs. (A) Population doubling time of cells during 2D expansion. The different shapes represent the individual passages (circle: low passage, i.e. P3, P5, and P7; square: high passage, i.e. P4, P6, and P8. (B) Average increase in number of cells per passage per donor. The colors represent the individual donors. GEE was used to determine statistical differences (Supplementary Table S4). * *P* ≤ 0.05, ** *P* ≤ 0.01, *** *P*≤ 0.005

Next, we assessed the proliferation capacity of the different cells by calculating the PDT at each passage. As shown in Figure 3A, the preserved hACs exhibited slightly lower population doubling times compared to the hACPCs and lesioned hACs, although the overall differences were minor (Supplementary Table S4). Similarly, the average increase in cell number per passage (Figure 3B) was comparable across all three cell types, indicating that they retained a stable proliferative capacity during *in vitro* expansion and thereby maintained their cellular phenotype.

### Human Induced Chondrocytes Derived Neo-Cartilage Upon Fibronectin-Based Selection

**Figure 4.**
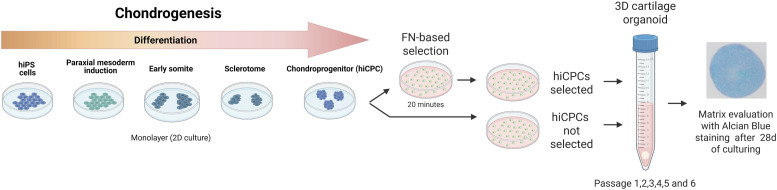
hiCPCs experimental set-up. HiPSCs were differentiated into hiCPCs though a stepwise differentiation protocol. At a defined stage (d14), cells were subjected to a FN adhesion assay selection for 20 minutes to enrich for chondroprogenitor populations based on their adhesive properties. hiCPCs selected and non-selected were then expanded in 2D culture up to six passages. At each passage, 3D cartilage organoids were generated and matrix production of matured human induced chondrocytes (hiCHOs) was evaluated through Alcian Blue staining after 28 days of maturation

HiPSCs were differentiated via a step-wise differentiation protocol into hiCPCs, which were then either subjected to FN-adhesion assay selection or left unselected before 2D expansion (Figure 4). 3D cartilage organoid cultures were generated from subsequent passages, whereas Alcian Blue staining was again used to assess cartilage matrix deposition and quality.

**Figure 5.**
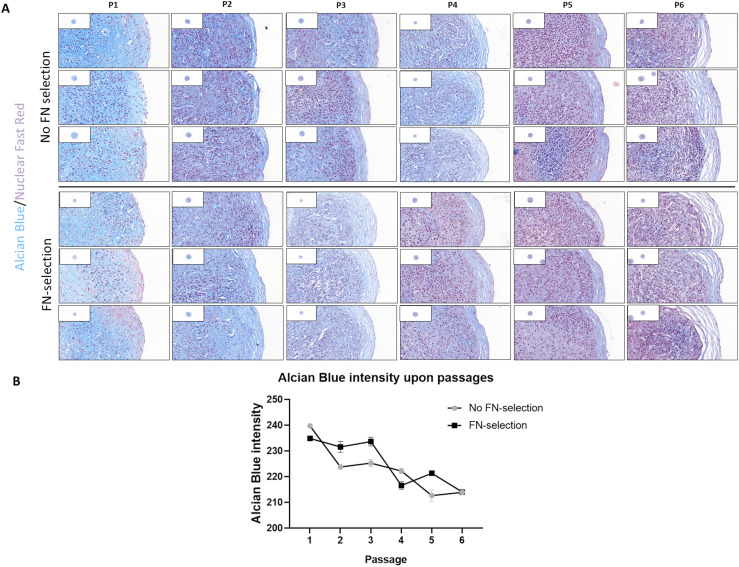
Alcian blue staining of hiPSC-derived chondrocytes (hiCHOs) 3D cartilage organoids in multiple passages, with or without FN adhesion assay selection. (A) Representative Alcian Blue/Nuclear Fast Red-stained sections. The overview images were captured at 4x magnification, while the zoomed-in images were taken at 20x magnification. (B) Quantification of Alcian Blue intensity upon passages. GEE was used to determine statistical differences (Supplementary Table S5)

Unselected hiCPC-derived organoids at passage 1, the stage typically used in our differentiation protocols, showed strong Alcian Blue staining, indicative of robust cartilage ECM deposition (Figure 5A, upper panel). However, staining intensity progressively declined with increasing passages, with the most pronounced drop occurring between passage 4 and passage 5 (Figure 5B, Supplementary Table S5). Overall, FN-selection of hiCPCs prior to cartilage organoid generation showed results comparable to unselected samples and did not enhance chondrogenic capacity during extended passaging (Figure 5A, lower panel), as evidenced by a similar decline in Alcian Blue staining intensity upon passaging (Figure 5B, Supplementary Table S5). To further assess extracellular matrix composition, additional histological and immunohistochemical staining was performed. Safranin-O/Fast Green staining and immunohistochemical analyses for collagen type II (COL2) and collagen type I (COL1) were conducted on hiCHO-derived organoids across passages, with corresponding quantitative intensity analyses (Supplementary Figures S2–S4, Supplementary Tables S6-S8). These analyses showed trends consistent with the Alcian Blue staining, with strong matrix deposition at early passages and a progressive reduction upon extended passaging. No clear differences were observed between FN-selected and unselected conditions. Notably, COL2 intensity decreased markedly over passages, while COL1 intensity, although also decreasing, remained consistently higher than COL2. To further quantify this shift in collagen composition, we calculated the normalized COL1/(COL1+COL2) ratio (Supplementary Figure S5, Supplementary Table S9). At early passages, unselected hiCHOs showed a slightly higher tendency for COL1 contribution compared to selected conditions, which may reflect differences in initial cell state rather than a sustained effect of FN-based selection, as this difference was not maintained during extended passaging. Overall, these findings indicate that, despite an initial variation, hiCHOs consistently maintain a relatively enriched COL1ECM upon passaging.

**Figure 6.**
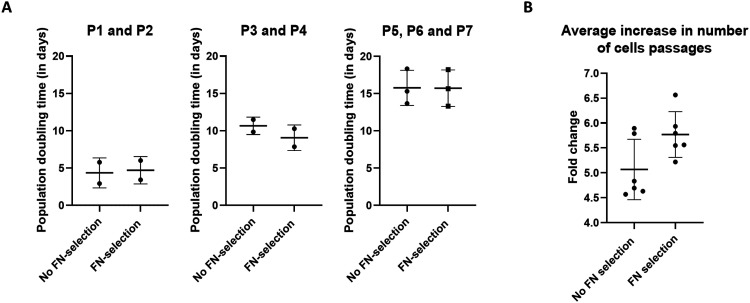
Population doubling time and increased cell numbers per passage of unselected hiCPCs and FN adhesion assay selected hiCPCs. (A) Population doubling time of cells during 2D expansion. (B) Average increase in number of cells per passage. GEE was used to determine statistical differences (Supplementary Table S10). * *P* ≤ 0.05, ** *P* ≤ 0.01, *** *P* ≤ 0.005

To evaluate whether FN-adhesion assay selection affected cell proliferation dynamics during serial passaging, we calculated the PDT at each passage. As shown in Figure 6A, FN adhesion assay selected cells exhibited PDTs comparable to those of unselected cells at all passages (Supplementary Table S10). Likewise, PDTs increased sharply under both conditions with successive passages, indicating changes in proliferative behavior that may reflect alterations in cellular state associated with extended passaging, consistent with the characteristics of the cartilage ECM shown in Figure 5A and Supplementary Figures S2-S4. Moreover, there was no significant difference in the average fold increase in cell number per passage between FN-selected and unselected cells (Figure 6B).

Overall, these findings suggest that the FN adhesion assay selection does not result in a more stable, progenitor-enriched hiCPC subpopulation capable of sustaining chondrogenic potential during extended passaging.

## Discussion

In this study, we demonstrated that both preserved and lesioned hACs maintained chondrogenic capacity across extended passaging and that hACPC-selection did not improve this chondrogenic potential. Similarly, FN-selection of hiCPCs failed to improve chondrogenic capacity, with Alcian Blue staining intensity declining and PDT increasing sharply after passage 3.

To compare the chondrogenic potential of preserved hACs, lesioned hACs and hACPCs, we generated 3D cartilage organoids at passage 2, 4, 6, and 8. Although we observed more variation in P8 compared to P2, all cells retained the capacity to produce cartilage matrix across passages, as shown by Alcian Blue staining and maintained structural organization at all evaluated time points. This contrasts with earlier studies reporting dedifferentiation and reduced matrix production following extended 2D expansion of primary chondrocytes.^19^ The sustained cartilage matrix production observed here, even from lesioned hACs, indicates that our isolation, expansion, and differentiation protocols effectively preserve the matrix-producing capacity of hACs. Comparison of our protocol with other published isolation and differentiation methods,^6,9,20,21^ revealed that chondrocyte isolation from cartilage is commonly performed using a two-step enzymatic digestion, typically combining pronase or hyaluronidase followed by collagenase, whereas our approach involves a single-step digestion using only collagenase. It is possible that this multi-step approach imposes excessive stress on the chondrocytes, potentially affecting their phenotype. In addition, considerable variation exists among chondrogenic differentiation protocols. In our approach, we supplement the culture medium with L-proline, sodium pyruvate and ascorbic acid, alongside commonly used components such as ITS+, dexamethasone, and TGF-β. These supplements are known to stimulate cartilage ECM production.^22-25^ Remarkably, lesioned hACs, despite being derived from visibly damaged cartilage, also demonstrated production of neo-cartilage constructs and structural integrity up to later passages. Their staining intensity and ECM organization were comparable to preserved counterparts, indicating that chondrocytes from lesioned areas retain functional potential despite originating from osteoarthritic tissue. This observation has important implications for autologous cartilage repair approaches, where donor tissue availability is often limited to lesioned regions.

Despite the widespread use of FN adhesion assays to enrich for progenitor-like cells which were reported to have enhanced proliferative capacity and chondrogenic potential compared to unselected cells,^6,8,9,19,26^ we did not observe improved chondrogenic potential when using FN-based selection to enrich for hACPCs. A recent study showed that the efficiency of FN-adhesion selection and subsequent chondrogenesis is highly dependent on the culture conditions, and the authors showed that FN together with fetal bovine serum actually reduced chondrogenic potential compared to serum-free conditions, suggesting complex interactions between FN, serum proteins, and chondrogenic fate.^27^ Other culture conditions that affect FN-based selection efficiency could be coating density, incubation time, and seeding density. A pilot experiment in which we varied the incubation time (5, 10 and 20 minutes) did not improve chondrogenic potential upon passaging (data not shown). Other parameters, including coating density and seeding density, were not systematically optimized in the current study, as we strictly followed the published protocol that showed beneficial effects of this selection.^28^

For hiCHOs, we employed a stepwise differentiation protocol to specifically obtain hiCPCs, which were then subjected to a FN adhesion assay or left unselected prior to 2D expansion and 3D neo-cartilage formation. This choice, targeting the progenitor stage at day 14 of differentiation rather than more differentiated hiCHOs, was deliberate, as previous work from our group has shown that this timepoint is particularly potent in starting chondrogenesis.^13,29-32^ Moreover, it also provided a clearer framework to test whether FN-based selection truly enriches for regenerative potential at an early, plastic stage of commitment. Overall, FN-based selection did not ultimately preserve the chondrogenic potential of the hiCPCs during extended passaging. From passage 4 onward, Alcian Blue staining declined sharply in both selected and unselected groups, in line with the previously observed dedifferentiation during extended 2D expansion.^12,17,18^

Several alternative strategies for generating chondroprogenitors from hiPSCs have been reported, yet key differences in chondrogenic potential and long-term stability remain insufficiently characterized. The advantage of our current step-wise differentiation protocol is that it has been optimized for the generation of hiCHOs, which closely resemble primary chondrocytes.^13^ Previously, we have also evaluated chondrogenic differentiation of hiPSCs via hiMSCs.^31^ Although hiMSCs maintain their phenotype over multiple passages, this approach resulted in more hypertrophic cartilage compared to our stepwise differentiation protocol and showed reduced similarity to hAC-derived cartilage. Kawata et al^33^ similarly reported chondrogenic differentiation from hiPSCs using two small-molecule compounds, with upregulation of chondrogenic markers and downregulation of pluripotency markers in their 2D cultures. However, no direct comparison to hAC-derived cartilage was performed, and the effects of serial passaging on chondrogenic potential were not assessed. Likewise, Smith et al^34^ introduced the RAPID protocol, which generates cartilage progenitors capable of forming high-quality cartilage, but without evaluation of long-term stability across passages. Taken together, these studies suggest that while multiple differentiation strategies can yield chondroprogenitors, key aspects such as maintenance of chondrogenic capacity over serial passaging remain insufficiently explored. Future studies should therefore investigate whether alternative differentiation strategies can generate more potent and stable chondroprogenitors that retain their chondrogenic potential over extended passaging.

A perceived weakness of our study is the fact that we mainly used Alcian Blue staining as quantitative read-out of chondrogenic capacity. This choice was intentional, as Alcian Blue has been shown to be a sensitive indicator of chondrogenic potential and, in our laboratory, demonstrates a strong correlation with cartilage molecular profiles.^4,29-31,35^ Moreover, Alcian Blue staining is widely recognized as a robust and reliable method for evaluating chondrogenesis. To gain additional insight into matrix composition, we included immunohistochemical analysis of collagen type I (COL1) in primary cell-derived neo-cartilage, and both COL1 and COL2, together with Safranin-O staining, in hiCHO-derived constructs.

For the primary cells, COL1 staining complemented the Alcian Blue findings by revealing subtle differences between cell sources. While all groups maintained matrix-forming capacity up to passage 8, cartilage derived from lesioned hACs showed a tendency toward higher COL1 levels, particularly at later passages. This is consistent with their origin from osteoarthritic tissue, where increased collagen type I deposition and a shift toward a more fibrocartilage-like phenotype are commonly observed.^4,29-31,35^ In contrast, preserved hACs and hACPCs displayed comparable COL1 levels, indicating stable matrix composition over time. FN-based selection did not alter these patterns, suggesting limited impact on collagen type I deposition in primary cells.

In hiCHO-derived matrix, analysis of collagen composition provided additional insight into the quality of the engineered cartilage. In native articular cartilage, collagen type II is the predominant fibrillar collagen, while collagen type I is minimal or absent, reflecting a hyaline cartilage phenotype.^4,29-31,35^ At early passages, hiCHO-derived neo-cartilage resembled this composition; however, this was not maintained over time. Upon passaging, a marked decrease in COL2 alongside a relatively higher contribution of COL1 was observed. A modest difference in the COL1/(COL1+COL2) ratio was present at early passages between FN-selected and unselected conditions, with unselected hiCHOs showing a slightly higher relative contribution of COL1 in the ECM; however, this difference was not maintained upon further passaging. These findings indicate that FN-based selection does not have a sustained effect on collagen composition and are consistent with dedifferentiation during in vitro expansion.^4,29-31,35^ These complementary approaches supported the trends observed across passages and reinforced the robustness of Alcian Blue as a primary readout. Due to technical constraints, additional stainings including SafO and COL2 could not be performed for the hACs and hACPCs. Nevertheless, integrating techniques such as quantitative biochemical assays for sGAG and collagen content, gene expression measurements and biomechanical characterization would further strengthen generalizability to evaluate tissue quality and functionality. Another potential weakness of our study is that we solely used a single healthy donor-derived hiPSC line, while it is known that hiPSC differentiation efficiency and chondrogenic potential can vary depending on donor-specific genetic and epigenetic backgrounds.^17^ However, our previous work has shown that key markers of cartilage quality, particularly those related to DNA methylation, remain relatively stable across different hiPSC lines during chondrogenesis.^13^ Nevertheless, to further improve the robustness of our findings, future studies would benefit from including multiple hiPSC lines.

In conclusion, FN adhesion–based selection of chondroprogenitor cells did not enhance chondrogenic capacity during extended passaging, either in primary cells or in hiPSC-derived cells. These results emphasize the importance of critically evaluating progenitor enrichment strategies for their functional benefits and support the use of unselected primary chondrocytes as a robust and practical cell source for cartilage tissue engineering.

## Supplemental Material

Supplemental Material - Comparative Assessment of Cartilage Quality in Human Induced Chondrocytes (hiCHOs) and Primary Articular Chondrocytes (hACs) Following Fibronectin-Based Selection Supplemental Material for Comparative Assessment of Cartilage Quality in Human Induced Chondrocytes (hiCHOs) and Primary Articular Chondrocytes (hACs) Following Fibronectin-Based Selection by Giorgia Mazzini, Margo Tuerlings, Vicki van der Stap, Ilja Boone, Rachid Mahdad, Yolande F.M. Ramos, and Ingrid Meulenbelt in CARTILAGE.

Supplemental Material - Comparative Assessment of Cartilage Quality in Human Induced Chondrocytes (hiCHOs) and Primary Articular Chondrocytes (hACs) Following Fibronectin-Based Selection Supplemental Materialfor Comparative Assessment of Cartilage Quality in Human Induced Chondrocytes (hiCHOs) and Primary Articular Chondrocytes (hACs) Following Fibronectin-Based Selection by Giorgia Mazzini, Margo Tuerlings, Vicki van der Stap, Ilja Boone, Rachid Mahdad, Yolande F.M. Ramos, and Ingrid Meulenbelt in CARTILAGE.

## Data Availability

Data is available upon request.*

## References

[1] Dilley JE Bello MA Roman N McKinley T Sankar U . Post-traumatic osteoarthritis: A review of pathogenic mechanisms and novel targets for mitigation. Bone Rep. 2023;18:101658. 37425196 10.1016/j.bonr.2023.101658 PMC10323219

[2] Bačenková D Trebuňová M Demeterová J Živčák J . Human Chondrocytes, Metabolism of Articular Cartilage, and Strategies for Application to Tissue Engineering. Int J Mol Sci. 2023;24(23):17096. 38069417 10.3390/ijms242317096 PMC10707713

[3] Takihira S Takao T Fujisawa Y , et al. Bioengineered chondrocyte-products from human induced pluripotent stem cells are useful for repairing articular cartilage injury in minipig model. npj Regenerative Medicine. 2025;10(1):31. 40593743 10.1038/s41536-025-00420-3 PMC12219148

[4] Bomer N den Hollander W Suchiman H , et al. Neo-cartilage engineered from primary chondrocytes is epigenetically similar to autologous cartilage, in contrast to using mesenchymal stem cells. Osteoarthritis and Cartilage. 2016;24(8):1423-1430. 26995110 10.1016/j.joca.2016.03.009

[5] Duan L Ma B Liang Y , et al. Cytokine networking of chondrocyte dedifferentiation in vitro and its implications for cell-based cartilage therapy. Am J Transl Res. 2015;7(2):194-208. 25901191 PMC4399086

[6] Williams R Khan IM Richardson K , et al. Identification and clonal characterisation of a progenitor cell sub-population in normal human articular cartilage. PLoS One. 2010;5(10):e13246. 20976230 10.1371/journal.pone.0013246 PMC2954799

[7] Rikkers M Korpershoek JV Levato R Malda J Vonk LA . The clinical potential of articular cartilage-derived progenitor cells: a systematic review. npj Regenerative Medicine. 2022;7(1):2. 35013329 10.1038/s41536-021-00203-6 PMC8748760

[8] Jiang Y Tuan RS . Origin and function of cartilage stem/progenitor cells in osteoarthritis. Nat Rev Rheumatol. 2015;11(4):206-212. 25536487 10.1038/nrrheum.2014.200 PMC5413931

[9] Rikkers M Korpershoek JV Levato R Malda J Vonk LA . Progenitor Cells in Healthy and Osteoarthritic Human Cartilage Have Extensive Culture Expansion Capacity while Retaining Chondrogenic Properties. Cartilage. 2021;13(2_suppl):129s-142s. 34802263 10.1177/19476035211059600 PMC8804833

[10] Jovic TH Thomson EJ Jones N , et al. Nasoseptal chondroprogenitors isolated through fibronectin-adherence confer no biological advantage for cartilage tissue engineering compared to nasoseptal chondrocytes. Frontiers in Bioengineering and Biotechnology. 2024:12-2024. 10.3389/fbioe.2024.1421111 PMC11464323 39391600

[11] Vinod E Parasuraman G Livingston A Sathishkumar S Ramasamy B . Isolation of Chondrocytes and Chondroprogenitors Using Fibronectin Adhesion and Migratory Assay. J Vis Exp. 2024;212:e67160. doi: 10.3791/67160. 39431785

[12] Adkar SS Wu CL Willard VP , et al. Step-Wise Chondrogenesis of Human Induced Pluripotent Stem Cells and Purification Via a Reporter Allele Generated by CRISPR-Cas9 Genome Editing. Stem Cells. 2019;37(1):65-76. 30378731 10.1002/stem.2931 PMC6312762

[13] Hajmousa G de Almeida RC Bloks N , et al. The role of DNA methylation in chondrogenesis of human iPSCs as a stable marker of cartilage quality. Clin Epigenetics. 2024;16(1):141. 39407288 10.1186/s13148-024-01759-y PMC11481477

[14] Ramos YF den Hollander W Bovée JV , et al. Genes involved in the osteoarthritis process identified through genome wide expression analysis in articular cartilage; the RAAK study. PLoS One. 2014;9(7):e103056. 25054223 10.1371/journal.pone.0103056 PMC4108379

[15] Dambrot C van de Pas S van Zijl L , et al. Polycistronic lentivirus induced pluripotent stem cells from skin biopsies after long term storage, blood outgrowth endothelial cells and cells from milk teeth. Differentiation. 2013;85(3):101-109. 23665895 10.1016/j.diff.2013.01.001

[16] Bomer N den Hollander W Ramos YF , et al. Underlying molecular mechanisms of DIO2 susceptibility in symptomatic osteoarthritis. Ann Rheum Dis. 2015;74(8):1571-1579. 24695009 10.1136/annrheumdis-2013-204739 PMC4516000

[17] Guzzo RM O’Sullivan MB . Human Pluripotent Stem Cells: Advances in Chondrogenic Differentiation and Articular Cartilage Regeneration. Current Molecular Biology Reports. 2016;2(3):113-122.

[18] Dicks A Wu C-L Steward N Adkar SS Gersbach CA Guilak F . Prospective isolation of chondroprogenitors from human iPSCs based on cell surface markers identified using a CRISPR-Cas9-generated reporter. Stem Cell Research & Therapy. 2020;11(1):66. 32070421 10.1186/s13287-020-01597-8 PMC7026983

[19] Kachroo U Vinod E . Comparative analysis of gene expression between articular cartilage-derived cells to assess suitability of fibronectin adhesion assay to enrich chondroprogenitors. The Knee. 2020;27(3):755-759. 32563433 10.1016/j.knee.2020.04.015

[20] van Mourik M Schuiringa GH Varion-Verhagen LP , et al. Enzymatic Isolation of Articular Chondrons: Is It Much Different Than That of Chondrocytes? Tissue Engineering Part C: Methods. 2022;29(1):30-40. 10.1089/ten.TEC.2022.0176 36576016

[21] Muhammad SA Nordin N Hussin P Mehat MZ Tan SW Fakurazi S . Optimization of Protocol for Isolation of Chondrocytes from Human Articular Cartilage. Cartilage. 2021;13(2_suppl):872s-884s. 31540551 10.1177/1947603519876333 PMC8804816

[22] Tee CA Roxby DN Othman R , et al. Metabolic modulation to improve MSC expansion and therapeutic potential for articular cartilage repair. Stem Cell Research & Therapy. 2024;15(1):308. 39285485 10.1186/s13287-024-03923-w PMC11406821

[23] Murdoch AD Hardingham TE Eyre DR Fernandes RJ . The development of a mature collagen network in cartilage from human bone marrow stem cells in Transwell culture. Matrix Biol. 2016;50:16-26. 26523516 10.1016/j.matbio.2015.10.003 PMC6042869

[24] Altaf FM Hering TM Kazmi NH Yoo JU Johnstone B . Ascorbate-enhanced chondrogenesis of ATDC5 cells. Eur Cell Mater. 2006;12:64-69. discussion 9–70. 17096313 10.22203/ecm.v012a08

[25] Shapiro IM Leboy PS Tokuoka T , et al. Ascorbic acid regulates multiple metabolic activities of cartilage cells. Am J Clin Nutr. 1991;54(6 Suppl):1209s-1213s. 1962572 10.1093/ajcn/54.6.1209s

[26] Pattappa G Reischl F Jahns J , et al. Fibronectin Adherent Cell Populations Derived From Avascular and Vascular Regions of the Meniscus Have Enhanced Clonogenicity and Differentiation Potential Under Physioxia. Frontiers in Bioengineering and Biotechnology. 2022;9:9-2021. 10.3389/fbioe.2021.789621 PMC8831898 35155405

[27] Basoli V Della Bella E Kubosch EJ Alini M Stoddart MJ . Effect of expansion media and fibronectin coating on growth and chondrogenic differentiation of human bone marrow-derived mesenchymal stromal cells. Scientific Reports. 2021;11(1):13089. 34158528 10.1038/s41598-021-92270-4 PMC8219706

[28] McCarthy HE Bara JJ Brakspear K Singhrao SK Archer CW . The comparison of equine articular cartilage progenitor cells and bone marrow-derived stromal cells as potential cell sources for cartilage repair in the horse. The Veterinary Journal. 2012;192(3):345-351. 21968294 10.1016/j.tvjl.2011.08.036

[29] Bloks NG Harissa Z Mazzini G , et al. A Damaging COL6A3 Variant Alters the MIR31HG-Regulated Response of Chondrocytes in Neocartilage Organoids to Hyperphysiologic Mechanical Loading. Advanced Science. 2024;11(36):2400720. 39021299 10.1002/advs.202400720 PMC11423154

[30] van Hoolwerff M Rodríguez Ruiz A Bouma M , et al. High-impact FN1 mutation decreases chondrogenic potential and affects cartilage deposition via decreased binding to collagen type II. Sci Adv. 2021;7(45):eabg8583. 34739320 10.1126/sciadv.abg8583 PMC8570604

[31] Rodríguez Ruiz A Dicks A Tuerlings M , et al. Cartilage from human-induced pluripotent stem cells: comparison with neo-cartilage from chondrocytes and bone marrow mesenchymal stromal cells. Cell Tissue Res. 2021;386(2):309-320. 34241697 10.1007/s00441-021-03498-5 PMC8557148

[32] Rodríguez Ruiz A van Hoolwerff M Sprangers S , et al. Mutation in the CCAL1 locus accounts for bidirectional process of human subchondral bone turnover and cartilage mineralization. Rheumatology (Oxford). 2022;62(1):360-372. 35412619 10.1093/rheumatology/keac232 PMC9788812

[33] Kawata M Mori D Kanke K , et al. Simple and Robust Differentiation of Human Pluripotent Stem Cells toward Chondrocytes by Two Small-Molecule Compounds. Stem Cell Reports. 2019;13(3):530-544. 31402337 10.1016/j.stemcr.2019.07.012 PMC6739881

[34] Smith CA Humphreys PA Naven MA , et al. Directed differentiation of hPSCs through a simplified lateral plate mesoderm protocol for generation of articular cartilage progenitors. PLoS One. 2023;18(1):e0280024. 36706111 10.1371/journal.pone.0280024 PMC9882893

[35] Tuerlings M Janssen GMC Boone I , et al. WWP2 confers risk to osteoarthritis by affecting cartilage matrix deposition via hypoxia associated genes. Osteoarthritis Cartilage. 2023;31(1):39-48. 36208715 10.1016/j.joca.2022.09.009

