# Supplementary material for: Comparative Assessment of Cartilage Quality in Human Induced Chondrocytes (hiCHOs) and Primary Articular Chondrocytes (hACs) Following Fibronectin-Based Selection
Source: Cartilage. 2026 Jun 8:19476035261458719. Online ahead of print. doi: 10.1177/19476035261458719 (PMC13246504; doi:10.1177/19476035261458719)
Supplement: Supplemental Material - Comparative Assessment of Cartilage Quality in Human Induced Chondrocytes (hiCHOs) and Primary Articular Chondrocytes (hACs) Following Fibronectin-Based Selection [file sj-pdf-1-car-10.1177_19476035261458719.pdf]

Supplementary file: tables S1-S10

Supplementary Table S1 *- Patient characteristics of three knee joints used to isolate hACPCs, preserved hACs and lesioned hACs*

	**Donor1**	**Donor2**	**Donor3**
Sex	Female	Male	Male
Age	80	79	57

Supplementary Table S2A*- Statistical difference in Alcian Blue staining intensity of primary cells derived neo-cartilage per passage, determined by GEE. Intensity ~ Passage(1|ID) and P2 is taken as a reference*

**Condition**	**Passage**	**B**	**Std. Error**	**95% Wald Confidence Interval**		**Hypothesis Test**	**Sig.**
				**Lower**	**Upper**	**Wald Chi-Square**	
hACs preserved	P4	3,82	1,34	1,19	6,45	8,09	**4,46E-03**
hACs preserved	P6	0,54	1,95	-3,28	4,35	0,08	7,83E-01
hACs preserved	P8	-1,75	1,20	-4,09	0,60	2,13	1,44E-01
hACPCs	P4	-7,24	3,50	-14,10	-0,38	4,28	**3,85E-02**
hACPCs	P6	-3,25	1,56	-6,30	-0,21	4,38	**3,65E-02**
hACPCs	P8	-19,34	13,15	-45,12	6,44	2,16	1,41E-01
hACs lesioned	P4	-1,22	2,69	-6,49	4,05	0,21	6,50E-01
hACs lesioned	P6	4,33	1,37	1,63	7,02	9,92	**1,64E-03**
hACs lesioned	P8	-6,04	2,15	-10,26	-1,83	7,91	**4,92E-03**

Supplementary Table S2B *- Statistical difference in Alcian Blue staining intensity of primary cells derived neo-cartilage, determined by GEE. Intensity ~ Condition(1|ID).*

**Passage**	**Comparison**	**B**	**Std. Error**	**95% Wald Confidence Interval**		**Hypothesis Test**	**Sig.**
				**Lower**	**Upper**	**Wald Chi-Square**	
P2	hACs preserved vs hACPCs	-1,47	1,13	-3,69	0,75	1,69	1,94E-01
P4	hACs preserved vs hACPCs	9,59	3,57	2,58	16,59	7,20	**7,28E-03**
P6	hACs preserved vs hACPCs	2,32	2,22	-2,03	6,67	1,09	2,96E-01
P8	hACs preserved vs hACPCs	16,13	13,16	-9,66	41,92	1,50	2,20E-01
P2	hACs lesioned vs hACPCs	-0,64	0,76	-2,13	0,85	0,70	4,02E-01
P4	hACs lesioned vs hACPCs	2,37	2,07	-1,69	6,43	1,31	2,52E-01

P6	hACs lesioned vs hACPCs	3,15	0,71	1,77	4,53	19,96	**7,92E-06**
P8	hACs lesioned vs hACPCs	6,01	6,62	-6,96	18,99	0,82	3,64E-01
P2	hACs preserved vs hACs lesioned	0,19	1,58	-2,90	3,29	0,02	9,02E-01
P4	hACs preserved vs hACs lesioned	-4,84	2,56	-9,85	0,17	3,59	5,81E-02
P6	hACs preserved vs hACs lesioned	3,98	1,79	0,48	7,48	4,98	**2,57E-02**
P8	hACs preserved vs hACs lesioned	-4,10	1,89	-7,80	-0,41	4,73	**2,97E-02**

14

Supplementary Table S3A- *Statistical difference in Collagen type-I staining intensity of primary cell derived neo-cartilage per passage, determined by GEE. Intensity ~ Passage(1|ID) and P2 is taken as [illegible] reference*

				95% Wald Confidence Interval		Hypothesis Test	
**Condition**	**Passage**	**B**	**Std. Error**	**Lower**	**Upper**	**Wald Chi-Square**	**Sig.**
hACs preserved	P4	2,28	1,99	-1,62	6,18	1,31	2.52E-01
hACs preserved	P6	-0,80	2,15	-5,02	3,41	0,14	7.09E-01
hACs preserved	P8	-8,90	6,50	-21,63	3,84	1,88	1.71E-01
hACPCs	P4	2,52	5,46	-8,18	13,21	0,21	6.45E-01
hACPCs	P6	-4,61	7,55	-19,41	10,19	0,37	5.41E-01
hACPCs	P8	-8,35	10,56	-29,04	12,34	0,63	4.29E-01
hACs lesioned	P4	-0,29	2,90	-5,97	5,39	0,01	9.21E-01

hACs lesioned	P6	1,04	2,40	-3,66	5,73	0,19	6.64E-01
hACs lesioned	P8	2,24	1,08	0,12	4,36	4,30	**3.82E-02**

Supplementary Table S3B *- Statistical difference in Collagen type-I staining intensity of primary cells derived neo-cartilage, determined by GEE. Intensity ~ Condition(1|ID).*

**Passage**	**Comparison**			**95% Wald Confidence Interval**		**Hypothesis Test**	
		**B**	**Std. Error**	**Lower**	**Upper**	**Wald Chi-Square**	**Sig.**
P2	hACs preserved vs hACPCs	0,79	6,55	-12,04	13,62	0,01	9.04E-01
P4	hACs preserved vs hACPCs	0,55	0,56	-0,54	1,64	0,98	3.21E-01
P6	hACs preserved vs hACPCs	4,6	2,97	-1,23	10,43	2,39	1.22E-01
P8	hACs preserved vs hACPCs	0,25	0,75	-1,23	1,72	0,11	7.45E-01
P2	hACs lesioned vs hACPCs	2,48	2,01	-1,47	6,42	1,52	2.18E-01
P4	hACs lesioned vs hACPCs	-0,33	4,69	-9,52	8,87	0	9.45E-01
P6	hACs lesioned vs hACPCs	8,13	3,92	0,44	15,82	4,29	**3.83E-02**
P8	hACs lesioned vs hACPCs	13,07	7,53	-1,68	27,82	3,02	8.23E-02
P2	hACs preserved vs hACs lesioned	-1,69	5,03	-11,55	8,17	0,11	7.37E-01
P4	hACs preserved vs hACs lesioned	0,88	4,78	-8,49	10,25	0,03	8.54E-01
P6	hACs preserved vs hACs lesioned	-3,53	1,46	-6,39	-0,67	5,87	**1.54E-02**

P8	hACs preserved vs hACs lesioned	-12,83	7,61	-27,74	2,08	2,84	9.17E-02

Supplementary Table S4 *- Statistical difference in population doubling time of primary cells in 2D, determined by GEE. PDT~ Passage(1|ID)*

Passage	Comparison	B	Std. Error	95% Wald Confidence Interval Lower	Upper	Hypothesis Test Wald Chi-Square	Sig.
P3andP4	hACs preserved vs hACPCs	2,56	0,94	0,71	4,40	7,39	**6,55E-03**
P5andP6	hACs preserved vs hACPCs	0,91	1,12	-1,28	3,09	0,66	4,17E-01
P7andP8	hACs preserved vs hACPCs	2,29	0,96	0,41	4,17	5,73	**1,67E-02**
P3andP4	hACs lesioned vs hACPCs	1,45	1,00	-0,51	3,41	2,11	1,46E-01
P5andP6	hACs lesioned vs hACPCs	-2,26	1,25	-4,71	0,19	3,27	7,07E-02
P7andP8	hACs lesioned vs hACPCs	-2,19	1,72	-5,56	1,18	1,63	2,02E-01

Supplementary Table S5A *- statistical difference in Alcian Blue staining intensity of hiCHO-derived neo-cartilage per passage, determined by GEE: Intensity ~ Passage(1|ID) and P1 is taken as a reference.*

Condition	Passage	B	Std. Error	95% Wald Confidence Interval Lower	Upper	Hypothesis Test Wald Chi-Square	Sig.
No selection	P2	-15,98	0,38	-16,72	-15,25	1815,35	**2,20E-16**
No selection	P3	-14,45	0,67	-15,76	-13,13	465,48	**2,20E-16**
No selection	P4	-17,33	0,75	-18,81	-15,85	527,21	**2,20E-16**
No selection	P5	-23,44	2,13	-27,61	-19,27	121,34	**2,20E-16**
No selection	P6	-24,86	0,12	-25,10	-24,62	41968,13	**2,20E-16**
FN selection	P2	-4,04	1,53	-7,03	-1,05	7,00	**8,16E-03**
FN selection	P3	-0,95	1,35	-3,60	1,70	0,49	4,82E-01
FN selection	P4	-19,02	0,85	-20,70	-17,35	497,27	**2,20E-16**
FN selection	P5	-14,60	0,75	-16,08	-13,12	375,87	**2,20E-16**
FN selection	P6	-18,20	0,52	-19,23	-17,18	1204,63	**2,20E-16**

Supplementary Table S5B *- statistical difference in Alcian Blue staining intensity of hiCHO-derived neo-cartilage, determined by GEE: Intensity ~ Condition(1|ID).*

Passage	Comparison	B	Std. Error	95% Wald Confidence Interval Lower	Upper	Hypothesis Test Wald Chi-Square	Sig.
P1	FN selection vs no selection	-4,90	0,32	-5,52	-4,27	235,22	**2,20E-16**
P2	FN selection vs no selection	7,77	1,07	5,67	9,87	52,59	**4,10E-13**
P3	FN selection vs no selection	8,36	1,05	6,30	10,41	63,68	**1,44E-15**
P4	FN selection vs no selection	-5,62	0,87	-7,33	-3,91	41,59	**1,13E-10**
P5	FN selection vs no selection	8,69	1,13	6,47	10,91	59,03	**1,55E-14**
P6	FN selection vs no selection	0,18	0,50	-0,79	1,16	0,14	7,12E-01

Supplementary Table S6 - *Statistical difference in Safranin-O staining intensity of hiCHO-derived neo-cartilage per passage, determined by GEE: Intensity ~ Passage(1|ID) and P1 is taken as a reference.*

Condition	Passage	B	Std. Error	95% Wald Confidence Interval Lower	Upper	Hypothesis Test Wald Chi-Square	Sig.
No selection	P2	-22,54	3,19	-28,79	-16,29	49,96	**2,20E-16**
No selection	P3	-39,59	0,51	-40,59	-38,60	6070,19	**2,20E-16**
No selection	P4	-35,21	3,65	-42,36	-28,05	93,02	**2,20E-16**
No selection	P5	-41,56	1,29	-44,08	-39,03	1040,26	**2,20E-16**
No selection	P6	-28,10	1,83	-31,68	-24,51	235,93	**2,20E-16**
FN selection	P2	-26,93	3,47	-33,72	-20,13	60,38	**2,20E-16**
FN selection	P3	-33,77	3,19	-40,02	-27,52	112,21	**2,20E-16**
FN selection	P4	-30,26	4,99	-40,03	-20,49	36,83	**2,20E-16**
FN selection	P5	-38,23	3,17	-44,44	-32,02	145,67	**2,20E-16**
FN selection	P6	-34,53	3,11	-40,62	-28,43	123,39	**2,20E-16**

Supplementary Table S7 - *statistical difference in COL2 staining intensity of hiCHO-derived neo-cartilage per passage, determined by GEE: Intensity ~ Passage(1|ID) and P1 is taken as a reference.*

Condition	Passage	B	Std. Error	95% Wald Confidence Interval Lower	Upper	Hypothesis Test Wald Chi-Square	Sig.
No selection	P2	-19,06	1,75	-22,49	-15,63	118,52	**2,20E-16**
No selection	P3	-23,44	2,12	-27,60	-19,27	121,68	**2,20E-16**

No selection	P4	-19,39	1,64	-22,61	-16,17	138,96	**2,20E-16**
No selection	P5	-23,22	2,48	-28,09	-18,35	87,38	**2,20E-16**
No selection	P6	-25,37	1,75	-28,80	-21,94	209,96	**2,20E-16**
FN selection	P2	-10,64	1,94	-14,44	-6,84	30,10	**2,20E-16**
FN selection	P3	-14,90	1,91	-18,64	-11,16	60,97	**2,20E-16**
FN selection	P4	-15,36	1,98	-19,24	-11,48	60,14	**2,20E-16**
FN selection	P5	-17,78	2,13	-21,95	-13,61	69,80	**2,20E-16**
FN selection	P6	-24,64	1,79	-28,15	-21,13	189,47	**2,20E-16**

Supplementary Table S8 *- statistical difference in COL1 staining intensity of hiCHO-derived neo-cartilage per passage, determined by GEE: Intensity ~ Passage(1|ID) and P1 is taken as a reference.*

				95% Wald Confidence Interval		Hypothesis Test	
**Condition**	**Passage**	**B**	**Std. Error**	**Lower**	**Upper**	**Wald Chi-Square**	**Sig.**
No selection	P2	-7,42	1,55	-10,45	-4,39	23,02	**2,20E-16**
No selection	P3	-9,37	2,42	-14,11	-4,63	15,03	**2,20E-16**
No selection	P4	-21,12	1,63	-24,32	-17,92	167,59	**2,20E-16**
No selection	P5	-16,52	1,88	-20,21	-12,83	76,96	**2,20E-16**
No selection	P6	-18,80	1,56	-21,86	-15,74	145,10	**2,20E-16**
FN selection	P2	-7,03	1,70	-10,35	-3,71	17,19	**2,20E-16**
FN selection	P3	-9,44	2,40	-14,14	-4,74	15,51	**2,20E-16**
FN selection	P4	-9,92	1,97	-13,78	-6,06	25,37	**2,20E-16**
FN selection	P5	-17,95	1,69	-21,26	-14,63	112,50	**2,20E-16**
FN selection	P6	-19,77	1,54	-22,78	-16,76	165,54	**2,20E-16**

Supplementary Table S9 *- statistical difference in COL1/COL1+COL2 staining intensity ratios of hiCHO-derived neo-cartilage per passage, determined by GEE: Intensity ~ Passage(1|ID) and P1 is taken as a reference.*

				95% Wald Confidence Interval		Hypothesis Test	
**Condition**	**Passage**	**B**	**Std. Error**	**Lower**	**Upper**	**Wald Chi-Square**	**Sig.**
No selection	P2	4,88	1,25	2,43	7,34	15,15	**9.91e-05**
No selection	P3	7,38	3,37	0,78	13,99	4,80	**2.85e-02**
No selection	P4	7,43	1,68	4,13	10,73	19,47	**1.02e-05**
No selection	P5	0,53	3,64	-6,60	7,66	0,02	8.84e-01
No selection	P6	7,19	2,21	2,86	11,51	10,62	**1.12e-03**
FN selection	P2	14,61	4,72	5,35	23,86	9,57	**1.98e-03**
FN selection	P3	17,94	1,60	14,80	21,07	125,74	**2.00e+16**
FN selection	P4	-0,52	1,27	-3,02	1,97	0,17	6.80e-01
FN selection	P5	9,64	3,60	2,59	16,70	7,17	**7.41e-03**
FN selection	P6	9,75	3,44	3,00	16,50	8,02	**4.64e-03**

Supplementary Table S10*- Statistical difference in population doubling time of hiPSC-derived chondroprogenitors in 2D, determined by GEE. PDT ~ Condition(1|ID).*

Passage	Comparison	B	Std. Error	95% Wald Confidence Interval Lower	95% Wald Confidence Interval Upper	Hypothesis Test Wald Chi-Square	Sig.
P1toP2	Selected vs Not selected	0,36	1,36	-2,31	3,02	0,07	7,93E-01
P3toP4	Selected vs Not selected	-1,60	1,04	-3,63	0,43	2,38	1,23E-01
P5toP6toP7	Selected vs Not selected	-0,05	1,60	-3,19	3,09	0,00	9,75E-01
