# Supplementary material for: Comparative Assessment of Cartilage Quality in Human Induced Chondrocytes (hiCHOs) and Primary Articular Chondrocytes (hACs) Following Fibronectin-Based Selection
Source: Cartilage. 2026 Jun 8:19476035261458719. Online ahead of print. doi: 10.1177/19476035261458719 (PMC13246504; doi:10.1177/19476035261458719)

Supplementary file: figures S1-S5

Supplementary Figure S1 | Collagen type I staining of hACPC-, preserved hAC- and lesioned hAC-derived 3D cartilage organoids in multiple passages. (**A**) Representative Collagen type-I/Haematoxylin-stained sections. The overview images were captured at 4x magnification, while the zoomed-in images were taken at 20x magnification. (**B**) Quantification of the Collagen type-I intensity. GEE was used to determine statistical differences (**Supplementary Table S10**).

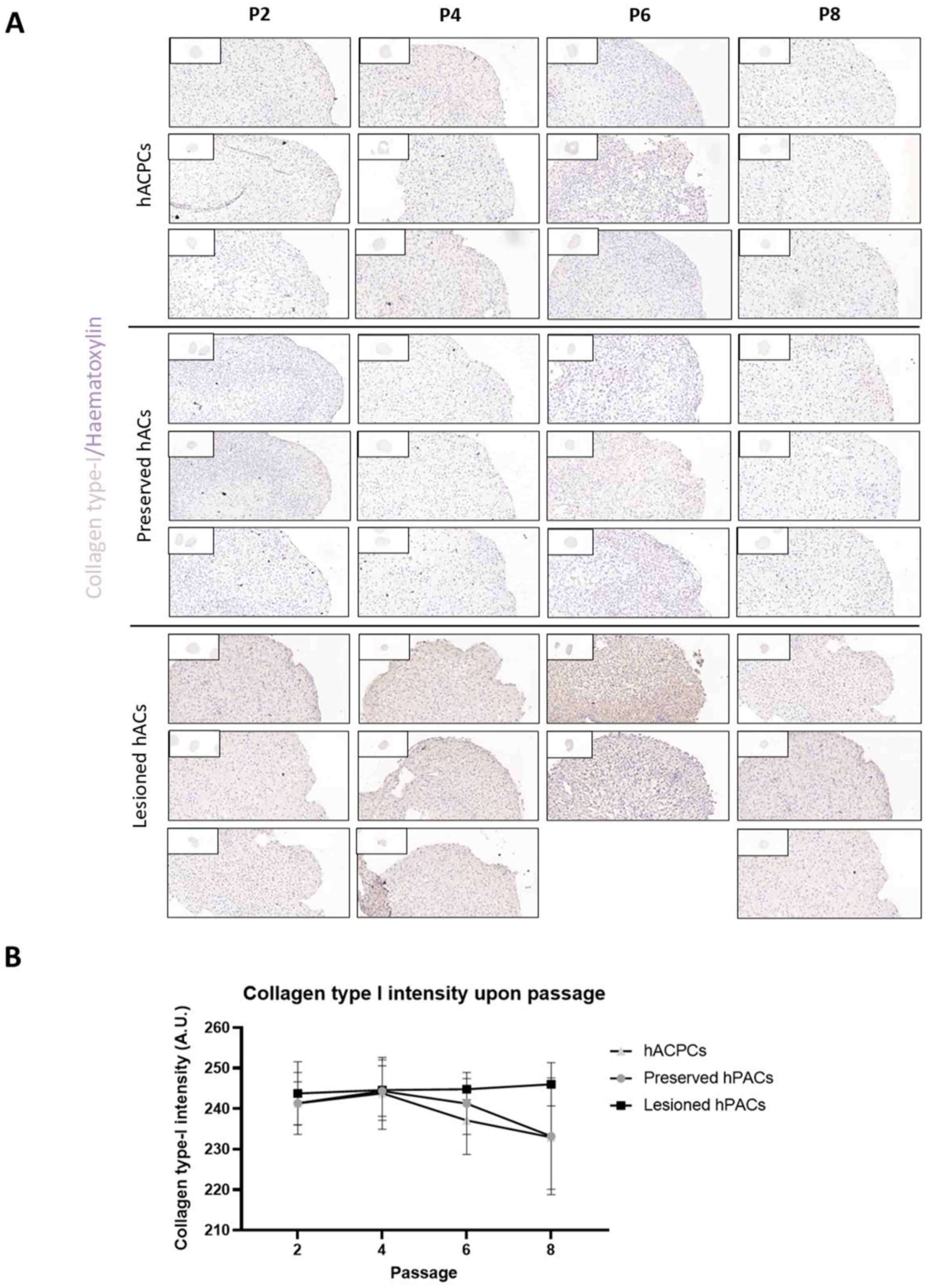

Supplementary Figure S2 | Safranin-O/Fast Green staining of hiPSC-derived chondrocyte (hiCHO) organoids across passages. (**A**) Representative sections of hiCHO-derived 3D cartilage organoids at passages p1–p6, with or without fibronectin (FN) selection. Safranin-O staining (red) indicates proteoglycan rich extracellular matrix, while Fast Green counterstains non cartilaginous components. The overview images were captured at 4x magnification, while the zoomed in images were taken at 20x magnification. (**B**) Quantification of the Safranin-O intensity upon passages (**Supplementary Table S6**).

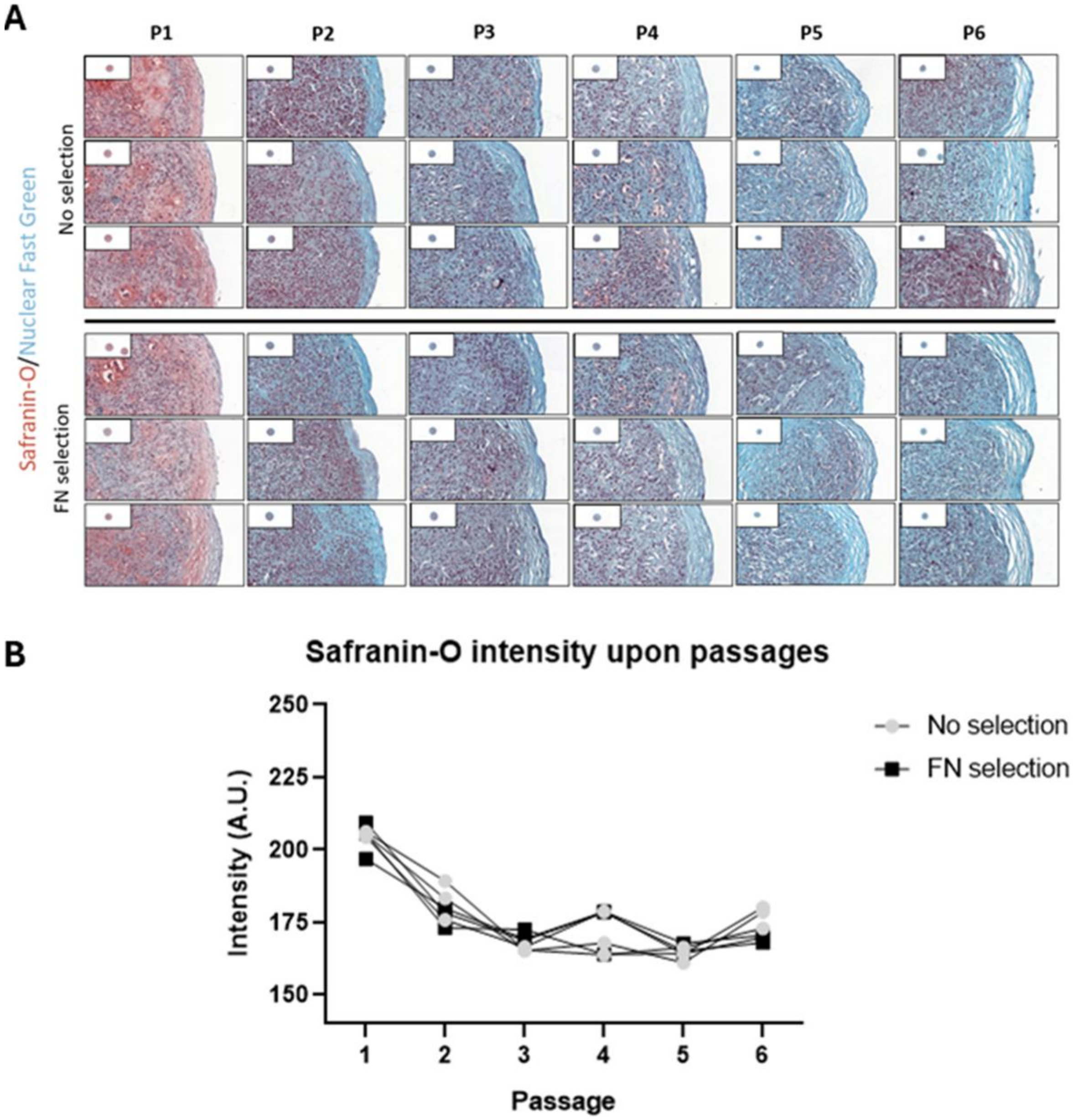

Supplementary Figure S3 | Immunohistochemical staining of collagen type II (COL2) in hiPSC-derived chondrocyte (hiCHO) organoids across passages. (**A**) Representative sections of hiCHO-derived 3D cartilage organoids stained for collagen type II (COL2) at passages p1–p6, with or without fibronectin (FN) selection. The overview images were captured at 4x magnification, while the zoomed in images were taken at 20x magnification. (**B**) Quantification of the COL2 intensity upon passages (**Supplementary Table S7**).

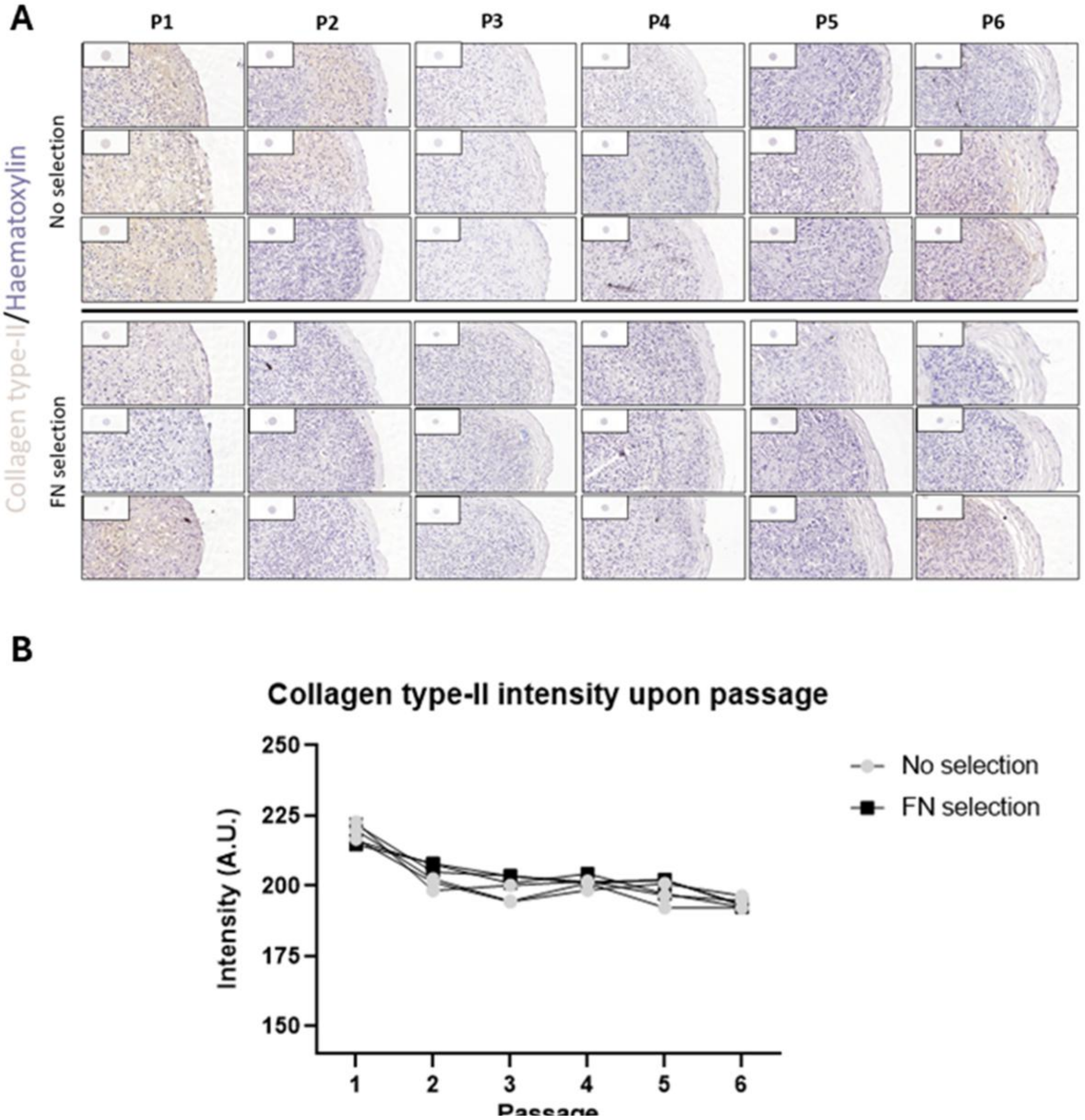

Supplementary Figure S4 | Immunohistochemical staining of collagen type I (COL1) in hiPSC-derived chondrocyte (hiCHO) organoids across passages. (**A**) Representative sections of hiCHO-derived 3D cartilage organoids stained for collagen type I (COL1) at passages p1–p6, with or without fibronectin (FN) selection. The overview images were captured at 4x magnification, while the zoomed in images were taken at 20x magnification. (**B**) Quantification of the COL1 intensity upon passages (**Supplementary Table S8**).

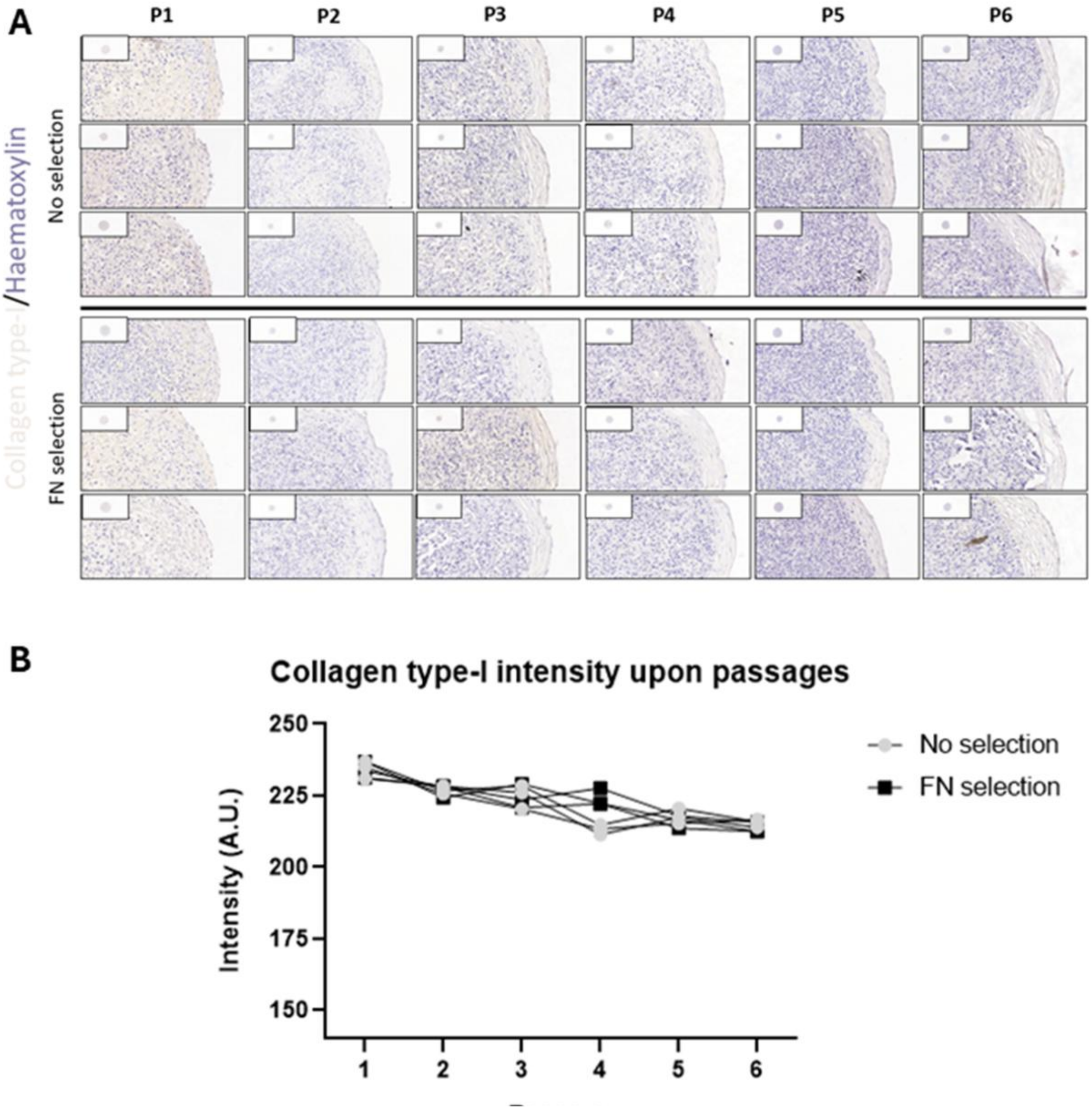

Supplementary Figure S5 | Quantification of COL1/(COL1+COL2) intensity ratio in hiPSC-derived chondrocyte (hiCHO) organoids across passages. The ratio between collagen type I (COL1) and collagen type II (COL2) staining intensities was calculated from immunohistochemical analyses at passages P1–P6, with or without fibronectin (FN) selection. GEE was used to determine statistical differences (**Supplementary Table S9**).

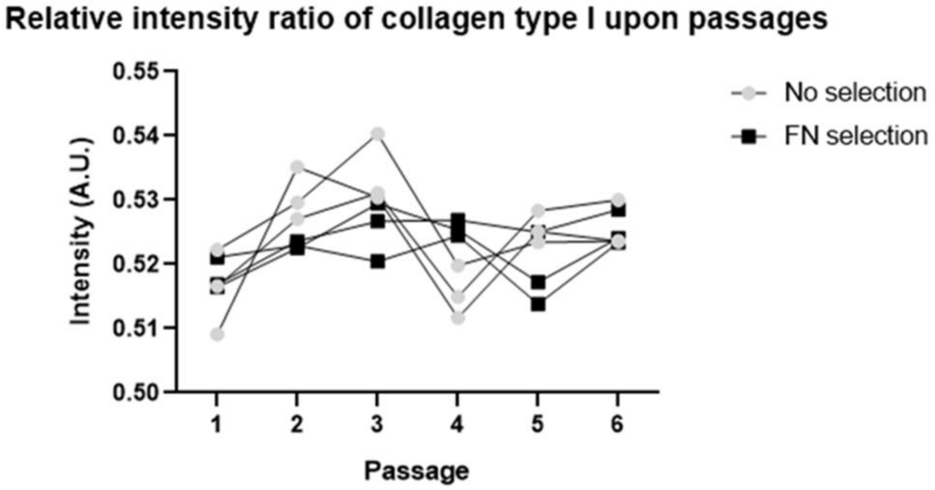

Supplement: Supplemental Material - Comparative Assessment of Cartilage Quality in Human Induced Chondrocytes (hiCHOs) and Primary Articular Chondrocytes (hACs) Following Fibronectin-Based Selection [file sj-pdf-2-car-10.1177_19476035261458719.pdf]
